# RNF2 inhibits E-Cadherin transcription to promote hepatocellular carcinoma metastasis via inducing histone mono-ubiquitination

**DOI:** 10.1038/s41419-023-05785-1

**Published:** 2023-04-11

**Authors:** Lei Yao, Jun Li, Bo Jiang, Zeyu Zhang, Xinying Li, Xiwu Ouyang, Yao Xiao, Guodong Liu, Zhiming Wang, Gewen Zhang

**Affiliations:** 1 Department of General Surgery, Xiangya Hospital, Central South University, No. 87, Xiangya Road, Changsha, 410008 China; 2 Department of thyroid surgery, First Affiliated Hospital of Zhengzhou University, No.1, East Construction Road, Zhengzhou, 450052 Henan China; 3 National Clinical Research Center for Geriatric Disorders, No. 87, Xiangya Road, Changsha, 410008 China

**Keywords:** Prognostic markers, Predictive markers, Liver cancer

## Abstract

RNF2 is a RING domain-containing E3 ubiquitin ligase that mediate histone H2A mono-ubiquitination to repress gene transcription, but its expression patterns and molecular function in hepatocellular carcinoma (HCC) remain unclear. Herein, we extracted data from TGCA database and validated RNF2 expression in our own cohort, which revealed that RNF2 was highly expressed in HCC and was associated with malignant characteristics and poor prognosis of HCC. Moreover, RNF2 was demonstrated to promote HCC metastasis via enhancing epithelial-mesenchymal transition (EMT) both in vitro and in vivo. Mechanistically, RNF2 repressed E-Cadherin transcription by increasing the deposition of H2K119ub at the E-Cadherin promoter region. In addition, RNF2-regulated crosstalk between H2AK119ub, H3K27me3 and H3K4me3 synergistically reduced E-Cadherin transcription, which promoted EMT and HCC metastasis. These results indicate that RNF2 played an oncogenic role in HCC progression via inducing EMT, and RNF2 could be a potential therapeutic target for HCC.

## Introduction

Hepatocellular carcinoma (HCC) is the sixth most prevalent malignancy in the world, accounting for more than 90% of primary liver cancer and is also the fourth leading cause of cancer-related mortality worldwide. [1, 2] Surgery to be the first-line treatment for HCC, with a 5-years survival rate over 70% for early-stage patients. [3, 4] However, because of the insidious onset of HCC, most patients are diagnosed at an advanced stage and have no chance of surgery, with a greatly reduced 5-year survival rate (<10%). [5] Therefore, it is necessary to develop new diagnostic markers and therapeutic targets for HCC.

RNF2 (also termed as Ring 1B), a major component of the poly-comb repressive complex 1 (PRC1), is a kind of RING domain-containing E3 ubiquitin ligase. RNF2 is highly expressed in multiple cancer types and contribute to tumor proliferation, metastasis, and drug resistance. [6–8] Wang et al. reported that RNF2 could catalyze the mono-ubiquitination of histone H2A at lysine 119 (H2AK119ub). [9] Meanwhile, previous works revealed a complex “cross-talks” between histone ubiquitination and methylation. [10–13] For example, the inhibition of H2A ubiquitination in mammals significantly reduced the recruitment of poly-comb repressive complex (PRC2) and H3K27me3 to the polycomb group (PcG) target. [14–16] Also, H2A ubiquitylation could inhibit transcription initiation by suppressing the dimethylation and trimethylation levels of H3K4. [10] However, the association between RNF2 and these histone modifications in HCC remained unclear.

During the multistage progression of neoplastic tumors, epithelial cells acquire a number of distinct mesenchymal features that confer on them the ability to invade adjacent tissues, and then spread to distant tissues. This phenotypic progression toward increased invasiveness is largely dependent on the activation of epithelial-mesenchymal transition (EMT). [17] Pathological reactivation of the EMT process plays a critical role in cancer metastasis. [17, 18] EMT is marked by multiple proteins including E-Cadherin, Claudins, N-Cadherin, etc, in which E-Cadherin is an important epithelial marker that mediates suppressive effects in tumor metastasis. [17, 19] Classical EMT occurs mainly through alternating expression of epithelial or mesenchymal genes. In cancer cells, post-translational modifications of EMT genes, such as covalent modifications of histones, can directly affect EMT gene expression and induce epithelial or mesenchymal phenotypes. [17] Previous studies revealed that histone modification at the E-Cadherin promoter region could affect the transcription of E-Cadherin to promote or suppress the metastasis of gastric cancer, lung cancer, and colon cancer. [20–22] However, the association between RNF2 and E-Cadherin in HCC remained unknown.

Herein, we comprehensively explored the prognostic value and biological function of RNF2 in HCC and revealed its association with histone modification, which would provide a new biomarker for HCC diagnosis and a novel target for HCC treatment.

## Results

### Upregulated RNF2 indicates poor prognosis of HCC patients

**Fig. 1:**
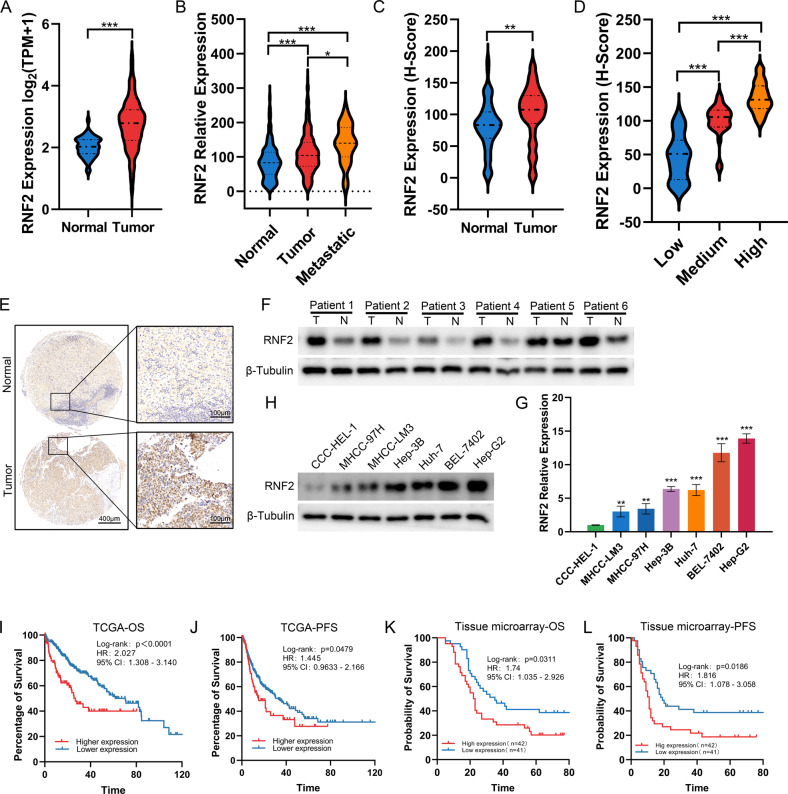
Upregulated RNF2 indicates poor prognosis of HCC patients. **A** The expression level of RNF2 in tumor and normal tissues was analyzed using TGCA-LIHC dataset. **B** By filtering and analyzing the data in TGCA-LIHC dataset, the expression of RNF2 in normal, tumor and metastasis showed that RNF2 was significantly up-regulated in tumor and metastasis, and the expression in metastasis was higher than that in tumor. **C** Analysis of RNF2 protein expression in 83 pairs of cancer and normal tissues by immunohistochemistry staining with tissue microarray showed that RNF2 was highly expressed in tumor. **D** According to the IHC H-Score of 83 HCC specimens, they were divided into low, medium and high groups. Analysis of the expression of RNF2 protein suggested that the expression of RNF2 gradually increased with the grouping. **E** Representative IHC staining images show RNF2 in normal and cancer tissues. **F** WB results showed the protein expression of RNF2 in 6 pairs of HCC patients’ surgical specimens (T: tumor, N: normal tissue). **G** Quantitative RT-PCR results revealed the expression levels of RNF2 in six HCC cell lines and normal liver cell line. **H** WB results showed the expression level of RNF2 in 6 HCC cell lines and normal liver cell line. **I**–**J** Kaplan-Meier analysis showed that HCC patients with high RNF2 expression in TGCA-LIHC dataset were significantly associated with lower OS and PFS. **K**–**L** Kaplan-Meier analysis showed that HCC patients with high RNF2 expression in our own cohort were significantly associated with lower OS and PFS.

To preliminarily explore the role of RNF2 in HCC, we extracted data from TCGA-LIHC dataset, which showed that RNF2 expression was significantly elevated in HCC tissues compared with normal tissues (Fig. 1A). Moreover, its expression was significantly increased in metastatic tumors (Fig. 1B). Similarly, in our own cohort composed of 83 HCC specimens, the protein level of RNF2 was significantly elevated in HCC tumor, and its expression was increased in high immunoreactivity score (IRS) group (Fig. 1C–E). Western blot confirmed the elevated expression of RNF2 in 6 paired HCC and normal specimens (Fig. 1F; Supplementary Fig. S9). Meanwhile, compared with normal hepatocytes (CCC-HEL-1), the mRNA and protein levels of RNF2 were significantly elevated in six HCC cell lines (Hep-G2, BEL-7402, Huh-7, Hep-3B, MHCC-97H, MHCC-LM3) (Fig. 1G, H; Supplementary Fig. S9).

As for its prognostic value, high expression RNF2 was significantly associated with poor overall survival (OS) and progression-free survival (PFS) in TCGA and our own cohorts (Fig. 1I–M). Based on RNF2 expression, we divided 83 HCC patients into high- and low-expression groups. High expression of RNF2 was positively correlated with microvascular invasion (MVI) (*p* = 0.019), number of tumor (*p* = 0.010) and advanced TNM stage (*p* = 0.039) (Table 1). Moreover, univariate Cox analysis identified AFP level (*p* = 0.012), number of tumor (*p* < 0.001), MVI (*p* < 0.001), TNM stage (*p* < 0.001) and RNF2 expression (*p* = 0.002) to be risk factors for HCC patients. In multivariate analysis, TNM stage and RNF2 expression were independent risk factors of OS (Table 2).

**Table 1:** Correlation analysis between RNF2 expression and clinical characteristics.

Variables	*n*	RNF2 expression		*p*
		Low	High	
Age (years)				0.161
≤48	31	18	13	
>48	52	23	29	
Gender				0.140
Female	75	39	36	
Male	8	2	6	
HBV infection				
Yes	67	33	34	0.588
No	16	8	8	
Cirrhosis				0.160
Yes	40	17	23	
No	43	24	19	
AFP				0.547
≤20	38	19	19	
>20	45	22	23	
Number of tumor				0.019
<3	74	39	35	
≥3	9	2	7	
Microvascular invasion				0.010
M0	58	34	24	
M1-2	25	7	18	
TNM stage				0.039
I	29	18	11	
II-III	54	23	31	
Pathology grade				0.105
II	32	18	14	
III	47	23	24	
IV	4	0	4

**Table 2:** Cox regression analysis of overall survival (OS) in 83 patients with HCC.

	Univariate analysis		Multivariate analysis	
	HR (95% CI)	*p*	HR (95% CI)	*p*
Age	0.997(0.975–1.019)	0.770	–	–
Gender	1.956(0.886–4.316)	0.097	–	–
HBsAg	2.487(1.173–5.273)	0.017	1.124(0.490–2.577)	0.782
Cirrhosis	1.247(0.743–2.092)	0.403	–	–
AFP	1.001(1.000–1.001)	0.012	1.000(1.000–1.001)	0.530
Number of tumor	1.948(1.383–2.744)	0.000	1.337(0.953–1.875)	0.093
MVI	4.421(2.521–7.755)	<0.001	1.450(0.780–2.698)	0.240
TNM stage	2.602(1.994–3.395)	<0.001	2.559(1.813–3.610)	<0.001
Pathology grade	1.516(0.934–2.462)	0.093	–	–
RNF2 expression	1.012(1.004–1.020)	0.002	1.013(1.004–1.022)	0.004

Since these clinical characteristics were closely related to tumor metastasis, we suspected that high expression RNF2 might contribute to HCC metastasis and result in poor prognosis.

### NR2C2 increases RNF2 transcription in HCC

**Fig. 2:**
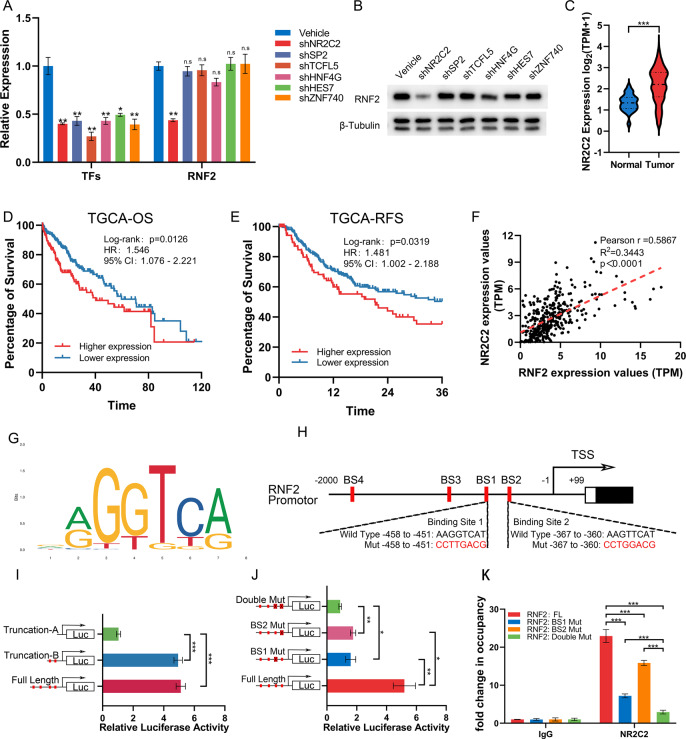
NR2C2 increases RNF2 transcription in HCC. **A**, **B** Quantitative PCR results and WB results revealed that only knockdown of NR2C2 down regulated the expression of RNF2, not the rest of TFs. **C** The expression level of NR2C2 in tumor and normal tissues was analyzed using TGCA-LIHC dataset. **D**, **E** Kaplan-Meier analysis showed that HCC patients with high NR2C2 expression in TGCA-LIHC dataset were significantly associated with lower OS and RFS. **F** Correlation analysis of TCGA-LIHC dataset showed that the expression of RNF2 was positively correlated with NR2C2. **G** The predicted NR2C2 binding motif found in the RNF2 promoter (JASPAR MA1536.1). **H** Possible binding sites of NR2C2 in RNF2 promoter region predicted by JASPAR database and the mutation strategy of BS1 and BS2. **I** Dual-luciferase reporter assay results of the full length and truncated RNF2 promoter sequence. **J** Dual luciferase reporter assay results of single mutation or double mutation of BS1 and BS2. **K** Chromatin immunoprecipitation (ChIP) was performed on 293 T cells transfected with different RNF2 promoter sequence vectors using anti-NR2C2 antibody, respectively.

To explore the reason of overexpressed RNF2 in HCC, we performed in silico analysis using UCSC genome browser and JASPAR database to search candidate transcription factors (TFs) that bound to the promoter region (from −2000 bp to +100 bp after the transcription start site) of RNF2 (Supplementary Table. 1). A total of six potential TFs were identified (JASPAR TBEs score≥500), and only silencing NR2C2 significantly downregulated RNF2 at mRNA and protein levels in Hep-G2 cells (Fig. 2A, B; Supplementary Fig. S9). NR2C2 was highly expressed in HCC tumors and indicated poor prognosis of HCC patients (Fig. 2C–E). Moreover, NR2C2 expression was positively associated with RNF2 in TCGA-LIHC dataset (Fig. 2F).

The binding motif for NR2C2 (JASPAR MA1536.1) was found in RNF2 promoter (Fig. 2G). Based on predicted binding sites (BS), we constructed wildtype and mutant luciferase vectors. Dual-luciferase reporter assay revealed that NR2C2 could bind to motifs near truncation-B to promote transcriptional activity (Fig. 2I). The mutation of single BS1 or BS2 significantly reduced luciferase activity whereas the double mutant BS1&BS2 significantly decreased luciferase activity (Fig. 2J). Further, chromatin immunoprecipitation (ChIP) assays confirmed that NR2C2 could bind to BS1 and BS2 of the RNF2 promoter (Fig. 2K).

These data suggested that NR2C2 could promote RNF2 expression via binding to its promoter region.

### RNF2 promotes invasion and migration of HCC via enhancing EMT

**Fig. 3:**
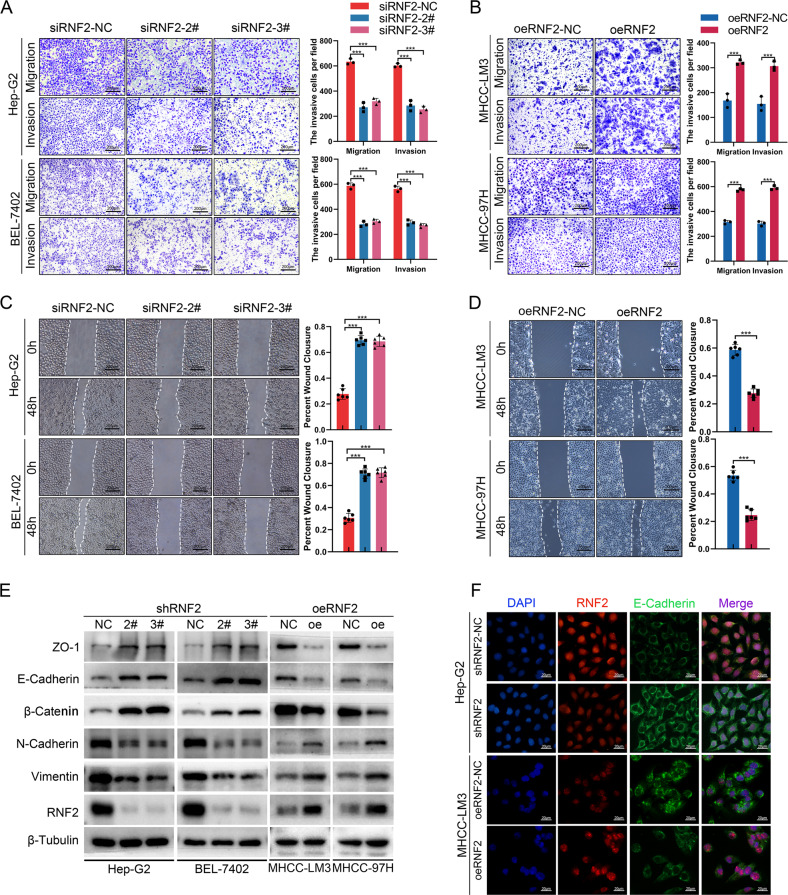
RNF2 promotes invasion and migration of HCC via enhancing EMT. **A**, **B** Representative images and quantitative analysis of the Transwell assay for transfected Hep-G2/BEL-7402/MHCC-LM3/MHCC-97H cells. **C**, **D** Representative images and quantitative analysis of the Wound healing assay for transfected Hep-G2/BEL-7402/MHCC-LM3/MHCC-97H cells. **E** Western blot assay results of protein level of EMT related molecules after silencing or overexpressing RNF2 in HCC cell lines. **F** Immunofluorescence (IF) images of the effect on E-Cadherin expression after knockdown of RNF2 in Hep-G2 cells or overexpression of RNF2 in MHCC-LM3 cells. Scale bar, 20 μm.

Then we investigated the function of RNF2 in HCC progression. Based on previous results, Hep-G2/BEL-7402 (with higher RNF2 expression) and MCHH-LM3/MHCC-97H (with lower RNF2 expression), were picked to perform the further study. Three siRNAs were designed to inhibit RNF2 expression and siRNA-2# and siRNA-3# were selected for their high efficiencies (Supplementary Fig. S1A, B; Supplementary Fig. S13). MTT assays indicated that the expression of RNF2 did not significantly affect the viability of HCC cells (Supplementary Fig. S2). However, transwell assays and wound healing assays revealed that the knockdown of RNF2 significantly reduced the migration and invasion of HCC cells (Fig. 3A, C). In contrast, the overexpression of RNF2 accelerated the migration and invasion of MHCC-LM3 and MHCC-97H cells (Fig. 3B, D; Supplementary Fig. S1C, D; Supplementary Fig. S13).

Previous studies indicated that epithelial mesenchymal transition (EMT) was a critical phenotype for tumor metastasis. [17, 18, 23] Therefore, we detected the expression levels of EMT-related molecules after RNF2 knockdown or overexpression in HCC cells. Results showed that the epithelial markers (ZO-1, E-Cadherin, β-Catenin) were increased and mesenchymal markers (N-Cadherin, Vimentin) were downregulated after RNF2 knockdown. The overexpressed RNF2 markedly decreased the expression of epithelial markers and increased the expression of mesenchymal markers (Fig. 3E; Supplementary Fig. S9). Moreover, as a transcription factor of RNF2, the effect of knocking down NR2C2 on the expression of EMT-related molecules was consistent with the results of shRNF2 (Supplementary Fig. S3; Supplementary Fig. S13). Furthermore, immunofluorescence (IF) revealed that RNF2 and E-Cadherin had opposite expression patterns (Fig. 3F).

Therefore, RNF2 was demonstrated to promote HCC metastasis via enhancing EMT.

### RNF2 promotes histone H2A ubiquitination to inhibit E-cadherin transcription

E-Cadherin epithelial cell adhesion protein is a tumor suppressor with an important role in tumor metastasis, and the absence of E-cadherin expression during epithelial-mesenchymal transition (EMT) is often thought to promote metastasis by allowing the isolation and invasion of cancer cells. [24] Previous studies have shown that H2AK119 mono-ubiquitination (H2AK119ub) was enriched in the E-Cadherin promoter region to suppress E-Cadherin expression and promote breast cancer metastasis. [25] Since RNF2 was a E3 ubiquitin ligase, we suspected that RNF2 might regulate H2AK119ub to promote the expression of E-Cadherin.

**Fig. 4:**
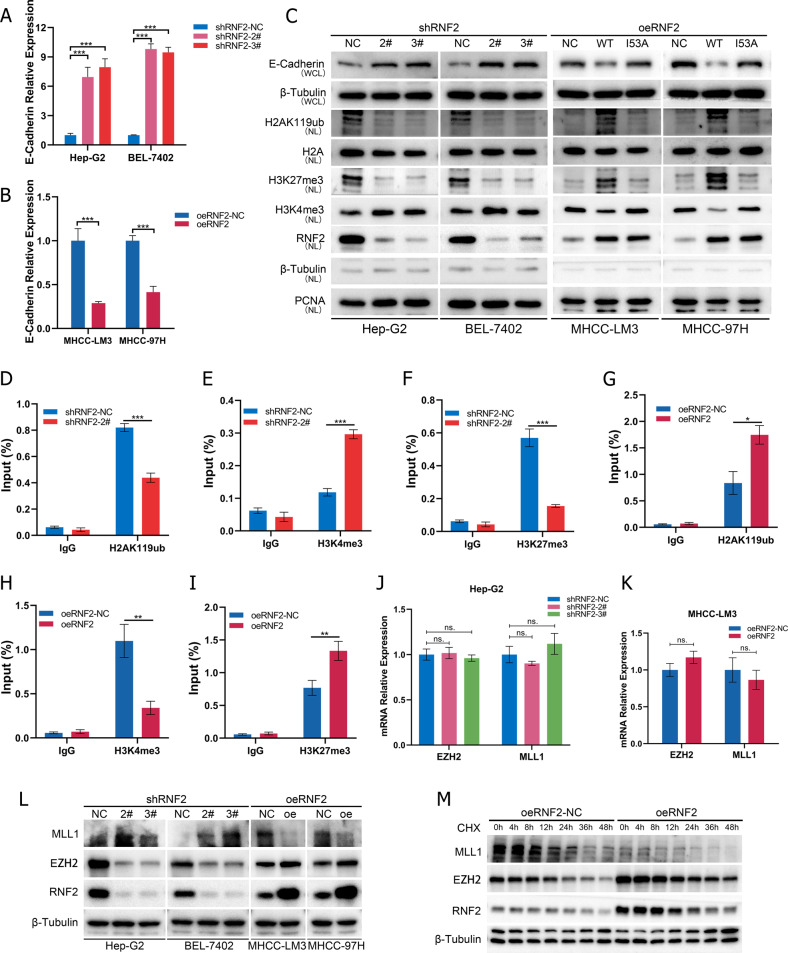
RNF2 promotes histone H2A ubiquitination to inhibit E-cadherin transcription. **A**, **B** Quantitative RT-qPCR results of altered E-Cadherin expression after knockdown or overexpression of RNF2 in HCC cell lines. **C** Western blot visualization of altered expression of H2K119ub, H3K27me3, H3K4me3 after knockdown/overexpression of RNF2 in HCC cell lines. WCL, whole-cell lysate; NL, nuclear lysate. **D**–**F** ChIP assay was performed on Hep-G2 cells transfected with shRNF2-NC/shRNF2-2# using anti-H2AK119ub, anti-H3K4me3 and anti-H3K27me3 antibodies, respectively. ChIP enrichments are normalized to input. **G**–**I** ChIP assay was performed on MHCC-LM3 cells transfected with oeRNF2-NC/oeRNF2 using anti-H2AK119ub, anti-H3K4me3 and anti-H3K27me3 antibodies, respectively. ChIP enrichments are normalized to input. **J**, **K** Quantitative RT-qPCR results of EZH2 and MLL1 expression after knockdown or overexpression of RNF2 in HCC cell lines. **L** Western blot visualization of altered expression of EZH2 and MLL1 after knockdown/overexpression of RNF2 in Hep-G2/MHCC-LM3 cell lines, respectively. **M** WB visualization of altered protein stability of EZH2 and MLL1 in MHCC-LM3 cell lines transfected with oeRNF2-NC/oeRNF2 after cycloheximide (CHX, 200 μM) treatment.

Firstly, we investigated the mRNA expression levels after transfected with lentivirus overexpressing or expressing shRNA specific for RNF2 in Hep-G2 and MHCC-LM3 cells, obviously, the expression of E-Cadherin increased in Hep-G2 cells and decreased in MHCC-LM3 cells, which is consistent with the protein level changes shown in the Fig. 3 (Fig. 4A, B). Based on these evidences, it is reasonable to assume that RNF2 directly affects the expression of E-Cadherin similar to the findings of Chen et al in pancreatic cancer. [26] We further investigated the expression and localization of RNF2 and E-Cadherin in HCC tissues and cells. IF staining revealed that RNF2 was mainly located in the nucleus while E-Cadherin was mainly located on the cell membrane (Supplementary Fig. S4). Meanwhile, the expression of E-Cadherin was higher in HCC than normal tissues, which differed from the expression pattern of RNF2 (Supplementary Fig. S4).

The knockdown of RNF2 significantly promoted the expression of E-Cadherin and H3K4me3 and decreased that of H2AK119ub and H3K27me3, whereas its overexpression had opposite effects (Fig. 4E; Supplementary Fig. S10). However, the mutation of ubiquitination site of RNF2 (oeRNF2-I53A) failed to exert oeRNF2-mediated effects (Fig. 4E; Supplementary Fig. S5; Supplementary Fig. S13). Moreover, ChIP assays revealed that the enrichment of H2AK119ub and H3K27me3 at E-Cadherin promoter was significantly reduced after RNF2 knockdown, whereas that of H3K4me3 was elevated (Fig. 4F–K; Supplementary Fig. 1L, M), and the overexpression of RNF2 had opposite effects. Similar results were detected that knockdown of NR2C2 promoted the level of H2AK119ub and H3K27me3 but decreased that of H3K4me3 (Supplementary Fig. S6). Previous studies revealed a crosstalk mechanism between H2A ubiquitination and H3K methylation, for instance, the increase of H2AK119ub positively affects the deposition of H3K27me3 but has the opposite effect on the deposition of H3K4me3, so we also added to measure their expression levels. [10, 11, 15, 27]

Further, we investigated how RNF2 modulate H3K27 and H3K4 trimethylations. Previous studies indicated that EZH2 and MLL1 were critical methyltransferase responsible for trimethylation of H3K27 and H3K4, respectively. [28–31] But, we found the altered expression of RNF2 did not affect the mRNA levels of EZH2 and MLL1 (Fig. 4J, K). However, the knockdown of RNF2 significantly reduced EZH2 expression but increased MLL1 expression, whereas overexpressed RNF2 exerted reversed effects (Fig. 4L; Supplementary Fig. S11). Meanwhile, the protein stability of MLL1 was reduced by RNF2 and that of EZH2 was promoted by RNF2 (Fig. 4M; Supplementary Fig. S11).

Taken together, these data demonstrated that RNF2 repressed E-Cadherin transcription by regulating H2AK119ub/H3K4me3/H3K27me3 at E-Cadherin promoter.

### E-Cadherin was involved in RNF2-mediated EMT in HCC cells

**Fig. 5:**
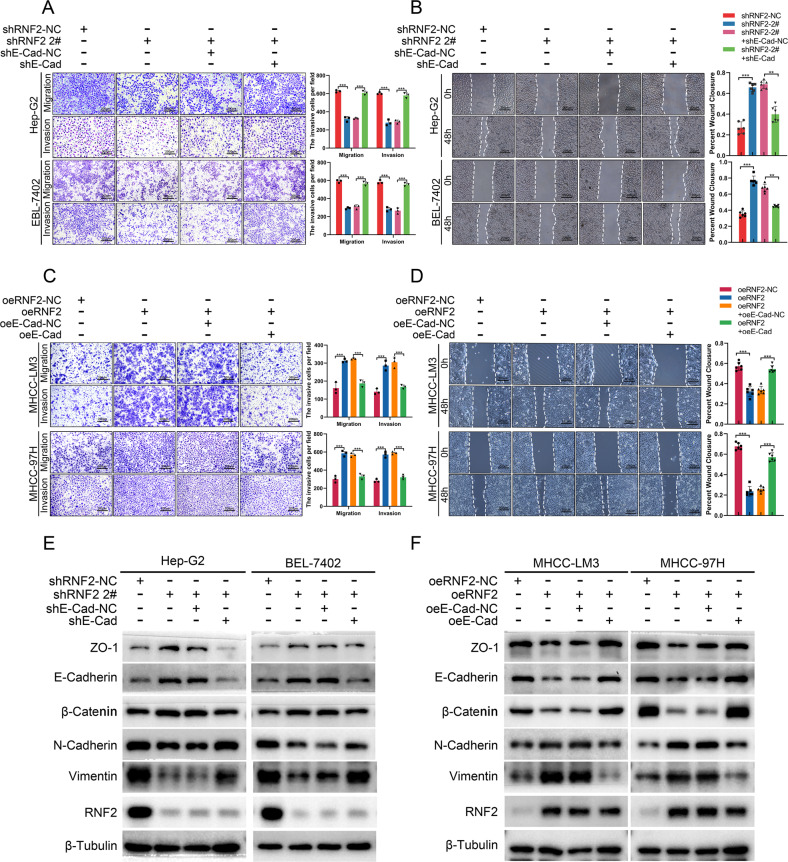
E-Cadherin was involved in RNF2-mediated EMT in HCC cells. **A**, **B** Representative images and quantitative analysis of Tanswell assay and Wound healing assay using transfected Hep-G2/BEL-7402 cells. **C**, **D** Representative images and quantitative analysis of Tanswell assay and Wound healing assay using transfected MHCC-LM3/MHCC-97H cells. **E** Western blot results of altered EMT-related protein expression after knockdown of RNF2 in Hep-G2/BEL-7402 cells and reversal of this alteration by co-knockdown of E-Cadherin. **F** Western blot results of altered EMT-related protein expression after overexpression of RNF2 in MHCC-LM3/MHCC-97H cells and reversal of this alteration by co-overexpression of E-Cadherin.

After identifying the interaction between RNF2 and E-Cadherin, we further explored whether E-Cadherin was involved in RNF2-mediated effects in HCC. The results showed that the invasive and migration of EBL-7402 and Hep-G2 were decreased by the knockdown of RNF2 but promoted by the inhibition of E-Cadherin (Fig. 5A, B; Supplementary Fig. S7). In contrast, the invasive and migration of MHCC-LM3 and MHCC-97H were enhanced by RNF2 but further reversed by E-Cadherin overexpression (Fig. 5C, D). Meanwhile, the expression of epithelial markers (ZO-1, E-Cadherin, β-Catenin) was increased and that of mesenchymal markers (N-Cadherin, Vimentin) was reduced by RNF2 knockdown, which was reversed by silenced of E-Cadherin (Fig. 5E; Supplementary Fig. S8; Supplementary Fig. S11; Supplementary Fig. S14). Similarly, RNF2-mediated effects on EMT markers were reversed by overexpressed E-Cadherin (Fig. 5F; Supplementary Fig. S12).

Therefore, these results demonstrated that RNF2 promoted EMT of HCC cells via regulating E-Cadherin expression.

### RNF2 inhibits E-Cadherin expression to promote HCC cell metastasis in vivo

**Fig. 6:**
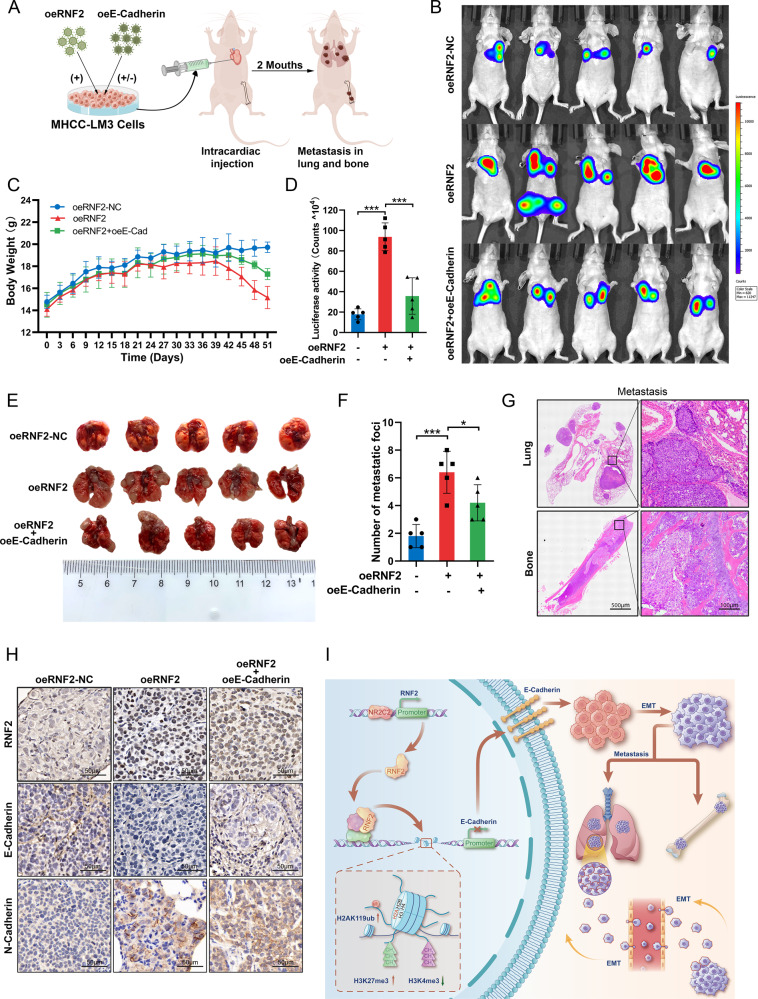
RNF2 inhibits E-Cadherin expression to promote HCC cell metastasis in vivo. **A** Schematic illustration of metastatic tumor model constructed in nude mice by intracardiac injection. **B** IVIS imaging system analyzes the fluorescence intensity in vivo. **C** Curves of body weight changes over time in nude mice after intracardiac injection of transfected cells. **D** Quantitative analysis of region of interests (ROI) counted by IVIS imaging system. **E**, **F** Representative images of lung metastasis in nude mice and quantitative analysis of the number of metastatic tumors. **G** Representative images of HE staining of mouse lung/bone metastasis gross anatomical specimens. **H** Representative IHC staining images of RNF2, E-Cadherin, and N-Cadherin expression levels in lung metastatic tumor tissues. **I** Illustration of the regulatory mechanisms of RNF2 in HCC.

Further, we validated the effects of RNF2 on HCC in vivo. The metastatic tumor model was established by intracardiac injection of HCC cells in nude mice (Fig. 6A). Compared with oeRNF2-NC group, the body weight of mice in oeRNF2 group was reduced and that of mice was elevated in oeRNF2+oeE-Cadherin group (Fig. 6C). Through bioluminescence imaging, we found that RNF2 promoted the metastasis of HCC cells and this was reversed after overexpression of E-Cadherin (Fig. 6B, D). After mice sacrifice, the number of metastatic nude in lung was significantly increased in oeRNF2 group, which was reduced by overexpressed E-Cadherin (Fig. 6E, F). IHC further confirmed that RNF2 expression was overexpressed in oeRNF2 group, E-Cadherin expression was reduced by RNF2 and promoted by overexpressed E-Cadherin, and N-Cadherin was promoted by RNF2 and decreased by overexpressed E-Cadherin (Fig. 6H).

Taken together, these results suggested that RNF2 could promote HCC metastasis via inhibiting E-Cadherin expression.

## Discussion

Intrahepatic and extrahepatic metastases are the main cause of death and poor prognosis of liver cancer. [32] In recent years, treatment strategies for unresectable HCC have been enriched with the development of systemic therapy. [33] However, due to the heterogeneity of HCC, the efficacy of targeted and immunotherapy cannot achieve optimal efficacy. [34, 35] Therefore, it is necessary to explore the effective diagnosis and prognosis biomarkers.

In this study, we demonstrated that RNF2 acts as an oncogene to promote HCC metastasis both in vitro and in vivo (Figs. 5, 6). Specifically, the high expression of RNF2 was directly regulated by NR2C2, which could bind to the promoter of RNF2 and enhance its expression (Fig. 2). RNF2 could increase the level of H2AK119ub at the E-Cadherin promoter region, which inhibited the transcription of E-Cadherin and promote HCC metastasis by inducing EMT (Fig. 4). Meanwhile, RNF2 could affect the stability of methyltransferase to promote H3K27me3 and reduce H3K4me3, which inhibited the transcription of E-Cadherin (Fig. 4).

Histone ubiquitination, a type of histone covalent modification, often occurs in histone H2A or H2B and is important for the regulation of chromatin dynamics and gene transcriptional. [9, 36, 37] PRC1 is a complex composed of more than eight different proteins, in which Ring1A/B confers E3 ubiquitin ligase activity, allowing it to specifically monoubiquitinate H2A at lysine 119. [9, 38] As the core subunit of the PRC1, Ring1B (RNF2) is widely reported as an oncogene in various cancers. In triple negative breast cancer, the deletion of RNF2 enhances the activation and infiltration of CD4 + -T and NK cells to promote anti-tumor immunity. [8] Overexpression of miR-139-5p in ovarian cancer cells suppresses RNF2 and MAPK-related cisplatin resistance. [39] Simultaneous silencing of Ring1A and Ring1B results in low levels of H2AK119ub modification, which inhibits Snail-mediated metastasis of pancreatic cancer cells. [26] Moreover, under chronic glucose stress conditions, RNF2 degrades HRIP via the ubiquitin proteasome pathway leading to apoptosis in breast cancer cells. [6] In present study, we found that RNF2 was highly expressed in HCC and was associated with malignant clinical features and poor prognosis (Fig. 1). Moreover, in vitro, transwell assays and wound healing assays confirmed that the expression levels of RNF2 were positively correlated with invasion and migration of HCC cells (Fig. 3). Furthermore, RNF2 could promote lung metastasis and bone metastasis of HCC cells in vivo (Fig. 6). Our study explored the expression pattern and prognostic value of RNF2 in HCC, which revealed a novel biomarker for HCC diagnosis. Meanwhile, RNF2 was identified as an oncogene that could be a potential target for HCC treatment.

EMT is a critical process for cancer metastasis, and its onset requires the cooperation of multiple molecular factors. [17, 18] Previous studies have shown that in HCC, the expression of EMT-related gene is regulated through transcriptional regulation, RNA methylation modification, DNA methylation modification, and ubiquitin-proteasome pathway, which in turn affect the metastatic ability of HCC cells. [32, 40–43] Here, we demonstrated that aberrant expression of RNF2 affected the expression of EMT-related proteins, which inhibited the expression of epithelial markers and but promoted that of mesenchymal markers (Fig. 3E). [43].

E-Cadherin, an important member of epithelium-associated molecules whose expression is regulated by multiple pathways and is frequently identified as a target gene for regulating EMT during tumor progression. [20–22, 25] In this study, immunofluorescence (IF) results showed that E-Cadherin expression was reduced in HCC and its expression was negatively associated with RNF2 expression (Fig. 3F; Fig. 4C, D). Moreover, E-Cadherin expression at the RNA level was also negatively regulated by RNF2 (Fig. 4A). In HCC cells, overexpressed E-Cadherin reversed the RNF2-induced EMT and subsequent promotion on HCC invasion and migration ability (Fig. 5A-D; Supplementary Fig. 2A, B). Therefore, we confirmed that RNF2 conferred oncogenic activities via regulating E-Cadherin in HCC.

Posttranslational modifications (PTMs) of histone are involved in epigenetic regulation that confers regulatory activities for gene expression, DNA replication and DNA repair. [27] PTMs include different kinds of covalent modifications such as phosphorylation, methylation, acetylation and ubiquitination. PRC1 and PRC2 are the two critical complexes responsible for histone modifications and there is a complicated crosstalk between different histone modification that regulate gene transcription. For example, H3K4me3 is an activated marker for gene transcription. PRC1 could catalyze H2AK119ub independent of PRC2 and H3K4me3. [28, 44] whereas H2A ubiquitination inhibits H3K4me3 and subsequently suppress transcription initiation. [10] Meanwhile, H3K27me3 is a repressive marker for gene transcription. The inhibition of H2A ubiquitination resulted in a significant decrease in PCR2 recruitment and H3K27me3 deposition on chromatin. [14, 45, 46] Herein, wild-type RNF2 significantly increased the level of H2AK119ub at E-Cadherin promoter region whereas mutant RNF2-I53A did not have similar effects (Fig. 4E). Moreover, RNF2-induced H2AK119ub increased the enrichment of H3K27me3 and decreased that of H3K4me3, which suppressed the transcription of E-Cadherin (Fig. 4E-K). We further investigated the mechanism by which H2AK119ub interacts with H3K27me3 and H3K4me3. EZH2 and MLL1 are the core methyltransferases that catalyze H3K27me3 and H3K4me3, respectively. [28, 47] Our results suggested that RNF2 could decrease the expression of EZH2 and MLL1 via mitigating their protein stabilities (Fig. 4L, M). Therefore, we speculated that RNF2 affect the expression of methyltransferase to promote H3K27me3 and decrease H3K4me3, which in turn reduce the transcription of E-Cadherin.

Another exciting discovery was the identification of NR2C2 as the transcriptional activator of RNF2. NR2C2 encodes a nuclear hormone receptor superfamily protein, which is known for its transcriptional activity and is involved in many biological processes, such as growth and development, meiotic, lipid metabolism, and the proliferation and maturation of erythroid cell. [48–52] Previous studies indicated an important role for NR2C2 in a variety of urological tumors. [53, 54] In this study, we found that NR2C2 is also highly expressed in HCC and correlated with poor prognosis (Fig. 2C–E). Through dual-luciferase reporter assay and ChIP experiments, we further demonstrated that NR2C2 could specifically bind to the BS1 and BS2 sites at the RNF2 promoter region and promote RNF2 transcription (Fig. 4H-K). These results firstly reveal the regulatory mechanism of highly expressed RNF2 in HCC, which provided a better understanding of the pathogenesis of HCC.

However, the way that RNF2 affected the stability of histone H3K27 and H3K4 methyltransferases remained unknown, and how RNF2-mediated H2AK119ub regulated H3K27me3 and H3K4me3 required additional investigation. The mutation of different domains of RNF2 is needed to confirm its impact on these PTMs.

In summary, our current work revealed that RNF2 played an oncogenic role in HCC progression via inducing EMT. RNF2-regulated crosstalk between H2AK119ub, H3K27me3 and H3K4me3 synergistically represses E-Cadherin transcription, which promoted HCC metastasis. These findings revealed that RNF2 contributed to HCC metastasis through regulating epigenetic modification and highlighted that RNF2 could be a potential therapeutic target and a prognostic marker for HCC.

## Methods and materials

### Patients and tissue specimens

For the analysis the protein expression level of the RNF2, 6 pairs HCC tumor tissue and adjacent normal tissues were freshly obtained from patients undergoing hepatectomy at Xiangya Hospital, Central South University (CSU). Ethical approval was approved by the ethics committee of Xiangya Hospital, Central South University (Grant No. 2019020053).

### Bioinformatic data collection

The transcriptome data and clinical information of TCGA-LIHC was downloaded from UCSC XENA (https://xenabrowser.net/), including 374 tumor samples and 50 normal samples.

### Cell culture and reagents

Hep-G2, MHCC-LM3, MHCC-97H, Huh-7 and Hep-3B were purchased from the Chinese Academy of Science Cell Bank (Shanghai, China). HEK-293T cells were kindly provided by Professor Cheng Ping Hu (Department of Respiratory Medicine, Xiangya Hospital, Central South University). CCC-HEL-1 and BEL-7402 cells were kindly provided by Dr. Yao Xiao (Department of General Surgery, Xiangya Hospital, Central South University). All cell lines were cultured in high-glucose DMEM medium (Hyclon, Logan, UT, USA) or RPMI-1640 medium (Hyclon, BEL-7402 cells only) supplemented with 10% fetal bovine serum (164210-500, ProCell, Wuhan, China), 100 U/mL penicillin, and 100 μg/ml streptomycin (15070063, Gibco) and incubated at 37 °C and 5% CO_2_.

### Protein extraction and western blotting

Whole-cell lysates were extracted using WB/IP Lysis Buffer (Beyotime, Shanghai, China) containing a protease inhibitor cocktail (Bimake, Houston, TX, United States). Nuclear protein was isolated using the Nuclear and Cytoplasmic protein extraction kit (Beyotime, Shanghai, China), according to the manufacturer’s instruction. All extracted proteins quantified using a BCA protein assay kit (Keygene, Nanjing, China) and using equal amounts of protein (20 µg) for gel electrophoresis. The protein lysates were separated by 10%/12% SDS PAGE gel electrophoresis and then transferred into PVDF membrane (Millipore). The PVDF membranes were then blocked with 5% skim milk for 1 h at room temperature and incubated with specific primary antibodies at 4 °C overnight. The HRP-conjugated anti-rabbit or anti-mouse IgG secondary antibodies were incubated 1 h at room temperature and the immunoblots were visualized using an enhanced chemiluminescence imaging system. The primary antibodies used in this study were listed in Supplementary Table 2.

### Quantitative real-time PCR (qRT-PCR)

Total RNA was extracted from cells using TRIzol reagent (Takara, Kyoto, Japan).

Reverse transcription was implemented using Evo M-MLV RT Kit with gDNA Clean for qPCR (Accurate Biology, #AG11728, Changsha, China). For RT-qPCR, 2x SYBR Green Pro Taq HS Premix II (Accurate Biology, #AG11701) was used and performed using the QuantStudio 5 real-time-PCR instruments (ThermoFisher, USA). Data were obtained from at least three independent experiments. Primer sequences used in this study were listed in Supplementary Table 3.

### Plasmid and transfection

NR2C2 and E-Cadherin overexpression plasmids and seven shRNAs (shNR2C2, shSP2, shTCFL5, shHNF4G, shHES7, shZNF740 and shE-Cadherin) were synthesized by General Biosystems (Anhui, China). The luciferase reporter plasmids, including full length and truncated RNF2 promoter wild-type plasmids and RNF2 promoter mutation plasmid were cloned into the pGL3 vector by General Biosystems (Anhui, China). All plasmid transfection experiments were performed using Lipofectamine-3000 (L3000015, Invitrogen, Eugene, OR, USA) according to the manufacturer’s protocols. NR2C2 and E-Cadherin overexpression vectors and packaging vectors were transfected into HEK-293T cells using Lipofectamine 3000 to produce lentivirus. The sequences of shRNA were listed in Supplementary table 4.

### Viruses and transduction

RNF2, RNF2-I53A (a Ring domain mutation of RNF2 makes it lack the ability to interact with E2 ligase) [55] overexpression and three sh-RNF2 lentiviru were obtained from GeneChem (Shanghai, China). Transfection was performed according to the manufacturer’s protocol, and puromycin (ST551, Beyotime, Beijing, China) was used to screen stably transfected cells.

### Transwell assay, wound healing assay and cell viability assay

Transwell cell migration/invasion assay and wound healing assay were implemented as described previously. [56] Briefly, 4 × 10^4^ cells (8 × 10^4^ cells for invasion assay) were resuspended in 200 μL FBS-free medium and seeded in the upper chamber, 600 μL culture media with 20% FBS was in the lower chamber. After 24/48 h incubation, cells in the upper layer of the chamber were washed away and migration or invasion cells in the lower layer were fixed in 4% paraformaldehyde at room temperature for 10 min and then stained in 0.01% crystal violet for 20 min, then photographed under a microscope. Data were obtained from at least three independent experiments. The wound healing assays were performed as described previously [57]. Cell viability was detected by MTT (Sigma) assay and performed as described previously [57].

### Chromatin immunoprecipitation (ChIP)

ChIP assay were performed using the Pierce™ Magnetic ChIP Kit (Thermo Fisher) according to the manufacturer’s instructions. Lentiviral transfected Hep-G2 and MHCC-LM3 cells was followed by cross-linking, uncross-linking and sonication fragmentation. The fragmented chromatin was collected and then immunoprecipitated with specific primary antibodies. DNA was extracted from the immunoprecipitates, and subjected to RT-PCR.

### Dual luciferase reporter assay

Dual luciferase reporter assay was conducted depending on the technical manual of Promega Dual-Luciferase Reporter Assay System. The firefly, Renilla luciferase reporter plasmid, and NR2C2 overexpression plasmid were co-transferred into HEK293 cells by Lipofectamine-3000. After 48 h, cells were lysed and luminescence intensity were measured.

### Tissue microarray and immunohistochemistry

For perform immunohistochemical (IHC) staining for RNF2, we purchased a tissue microarray slide from Servicebio (LVC-1608; Servicebio, consisting of 83 pairs of hepatocellular carcinoma tissue and adjacent normal tissues). Tumor tissue sections from nude mice were processed according to standard protocols and routinely examined by H&E staining. Firstly, the tissue sections were heated at 60 °C for 2 h, and then dewaxed with an alcohol gradient treatment. Then blocking the slides with 3% normal sheep serum (ZSbio, Beijing, China) for 1 h at room temperature. After blocking, specimens will be incubated overnight at 4 °C with primary antibody. The VECTASTAIN Elite ABC HRP kit (Vector Laboratories, Burlingame, CA, USA) and the VECTOR DAB kit (Vector Laboratories) were then used to color development according to the manufacturer’s instructions. The slides were visualized using Pannoramic Digital Slide Scanners (3DHISTECH, Budapest, Hungary).

### Immunofluorescence

Cells to be tested were inoculated with 24-well plates and cultured overnight, then fixed with formaldehyde at −20 °C for 20 min and permeabilized with 0.1% Triton X-100. Blocking cells with 3% normal sheep serum (ZSbio, Beijing, China) for 1 h at room temperature, then cells will be incubated with primary antibodies overnight at 4 °C. Next day, incubate the fluorophore-conjugated secondary antibody at room temperature. Fluoroshield mounting medium with DAPI (Abcam) was used for nuclei labeling. Fluorescence images are acquired by a multi-channel fluorescence microscope (Nikon, Japan).

### In vivo metastasis assay

Metastatic tumor model in nude mice was established by intracardiac injection as described previously. [58, 59] Female nude mice at 4–6 weeks of age, purchased from Hunan SJA Laboratory Animal Co. Ltd (Changsha, Hunan, China) were used for this experiment. Briefly, the mice were anesthetized by isoflurane inhalation, and the chest was routinely disinfected with 10% povidone-iodine solution and 70% ethanol, twice. Then 100 µl of resuspended MHCC-LM3 cells were extracted from a 1 ml insulin syringe, and slowly insert vertically along the slightly left of the midpoint between the sternal notch and the cartilago ensiformis, stop puncture when a red blood pulse is observed in the syringe and maintain this depth, and gently press down on the syringe to inject the cell suspension into the heart. A total of 5 × 10^5^ MHCC-LM3 cells/mouse were injected immediately after resuspension with PBS. After observing significant weight loss, D-fluorescein potassium salt was injected in intraperitoneally before isoflurane anesthesia. Fluorescent images in nude mice captured and analyzed by an IVIS imaging system (PerkinElmer, Waltham, MA, USA). Animal experiments were approved by the Animal Ethics Committee of Hunan SJA Laboratory Animal Co. Ltd (Grant No. SJ A2021018) and performed the Guidelines for the Care and Use of Laboratory Animals at Central South University.

### Statistical analysis

Statistical analysis of the data involved in the study was performed using SPSS 19.0 and GraphPad Prism 8.0. Independent t-test were used for comparisons between groups for continuous variables, and χ2 test were used for comparisons for categorical variables. The overall survival and disease-free survival curves of patients involved in tissue microarrays were plotted using the Kaplan-Meier method. Spearman rank correlation analysis was used to analyze the correlation between protein expression levels of RNF2 and the clinical characteristics of HCC patients. Univariate cox regression analysis was used to assess prognostic factors, and then these factors were entered into univariate cox-regression analysis. A *p* < 0.05 was considered significant difference between the groups (n.s, *p < 0.05, **p < 0.01, ***p < 0.001).

## Supplementary information

Supplementary Figures

Supplementary table 1

Supplementary table 2

Supplementary table 3

Supplementary table 4

Original data files

Author Contribution Statement

aj-checklist

## Data Availability

All data generated and/or analyzed during this research are included in this published article and its supplemental materials.
