# Supplementary material for: RNF2 inhibits E-Cadherin transcription to promote hepatocellular carcinoma metastasis via inducing histone mono-ubiquitination
Source: Cell Death Dis. 2023 Apr 11;14(4):261. doi: 10.1038/s41419-023-05785-1 (PMC10085990; doi:10.1038/s41419-023-05785-1)
Supplement: Supplementary file 1 — Supplementary Figures [file 41419_2023_5785_MOESM1_ESM.pdf]

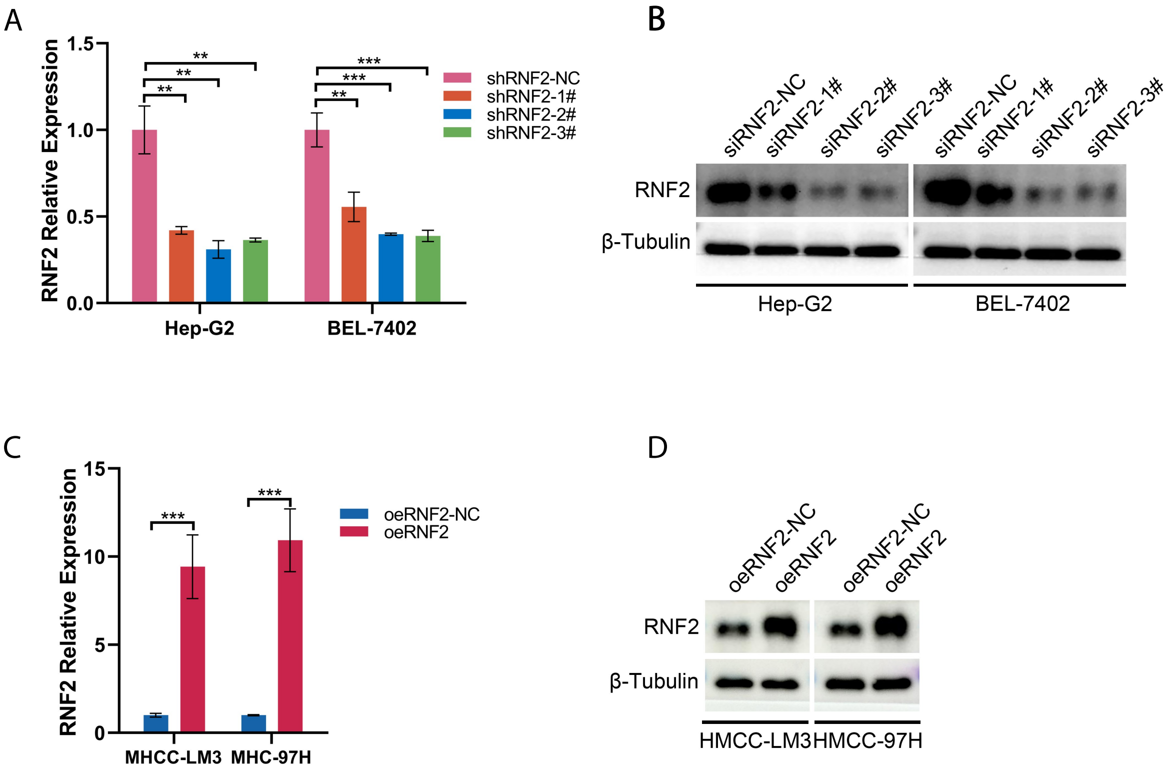

Supplementary Fig. S1 Knocdown and overexpression RNF2 in HCC cell lines.

(A, C) Quantitative qPCR analysis of knockdown and overexpression efficiency of RNF2 in HCC cell lines.

(B, D) WB results of knockdown and overexpression efficiency of RNF2 in HCC cell lines.

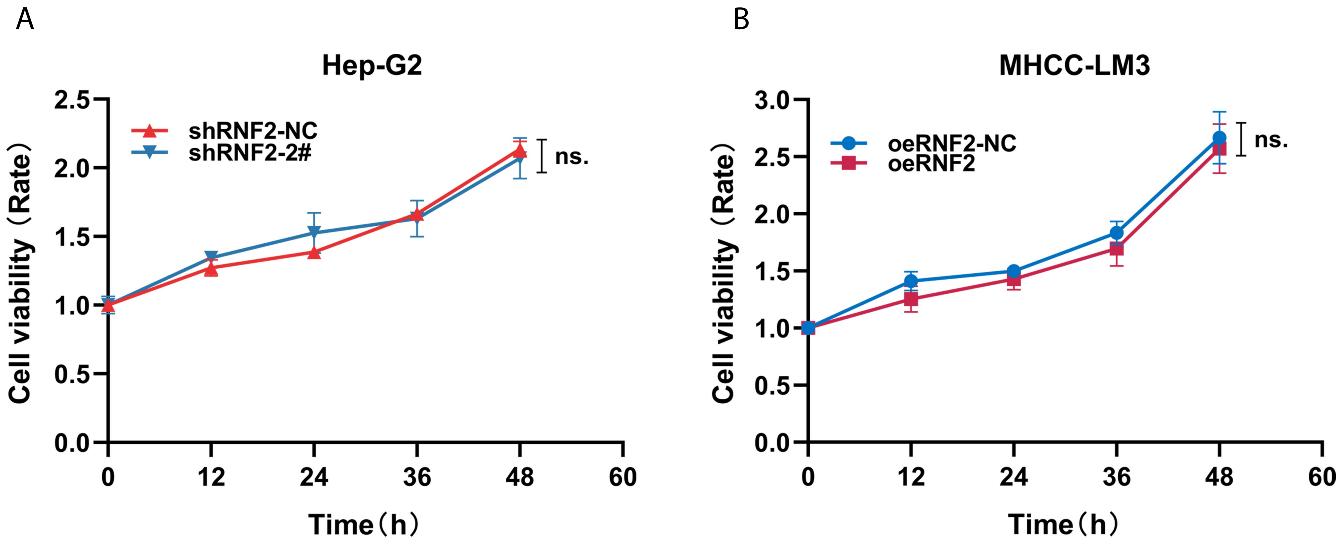

Supplementary Fig. S2 Cell viability of transfected Hep-G2 and MHCC-LM3 cells evaluated by MTT assays.

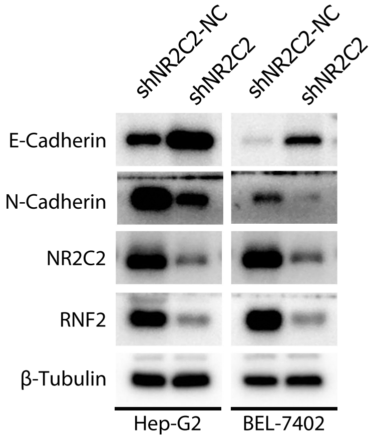

Supplementary Fig. S3 Western blot results of altered EMT-related protein expression after knockdown of NR2C2 in Hep-G2/BEL-7402 cells.

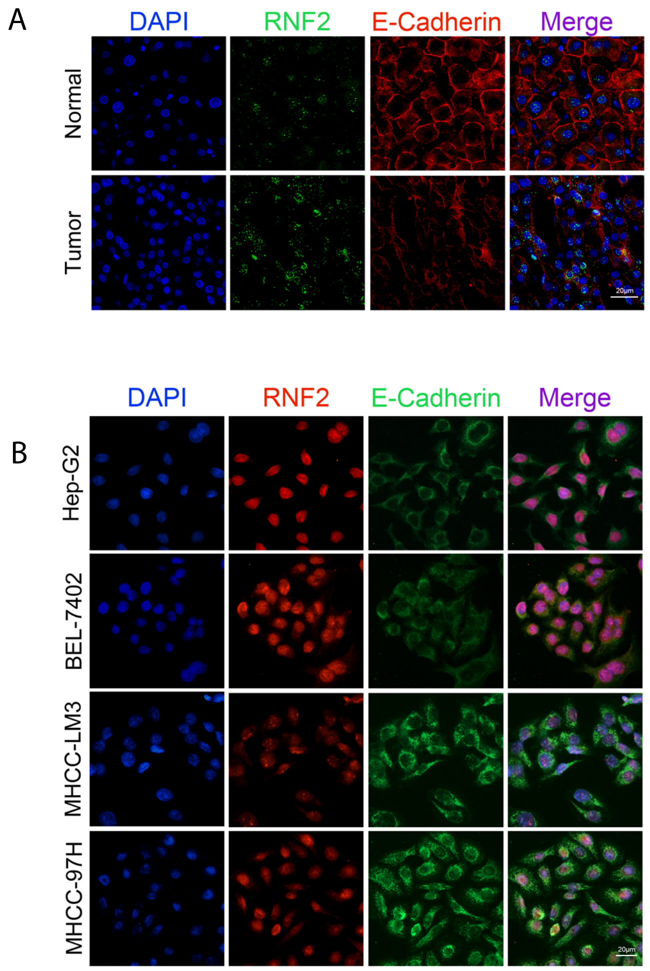

Supplementary Fig. S4 IF staining of the relationship between RNF2 and E-Cadherin.

(A) IF images of the pathological histological sections of tumors and normal tissues from HCC patients.

(B) IF images of the HCC wild-type cell lines. Scale bar, 20μm.

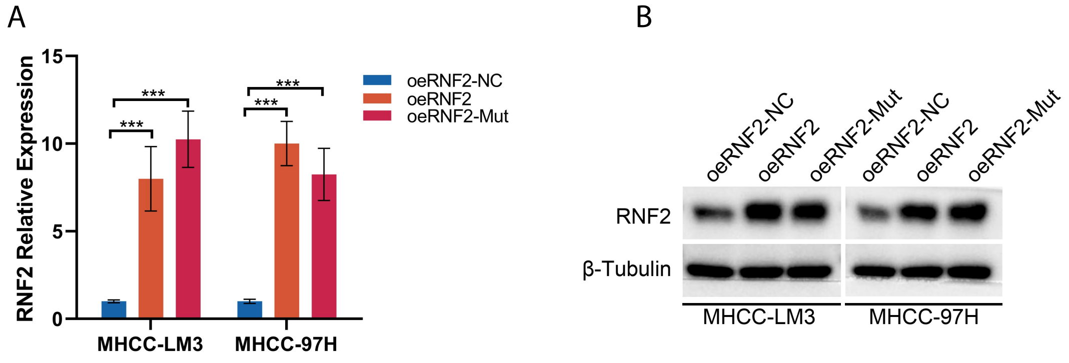

Supplementary Fig. S5 Overexpression wild-type and mutant RNF2 in HCC cell lines.

Quantitative qPCR analysis results (A) and WB results (B).

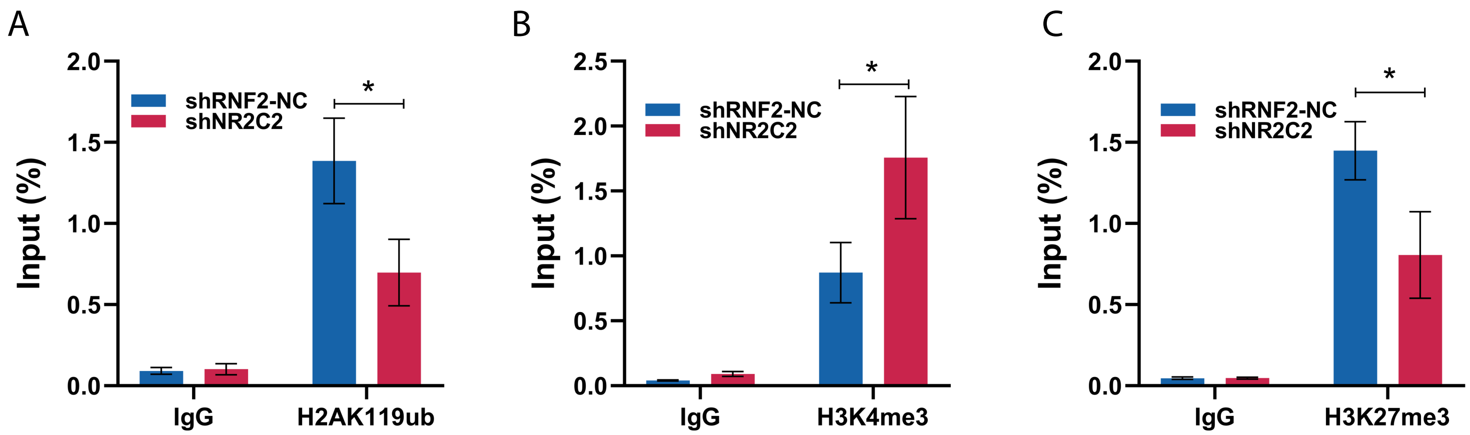

Supplementary Fig. S6 ChIP assay results of E-Cadherin promoter investigated by qPCR in Hep-G2 cells transfected with shRNF2-NC/shNR2C2 using anti-H2AK119ub, anti-H3K4me3 and anti-H3K27me3 antibodies.

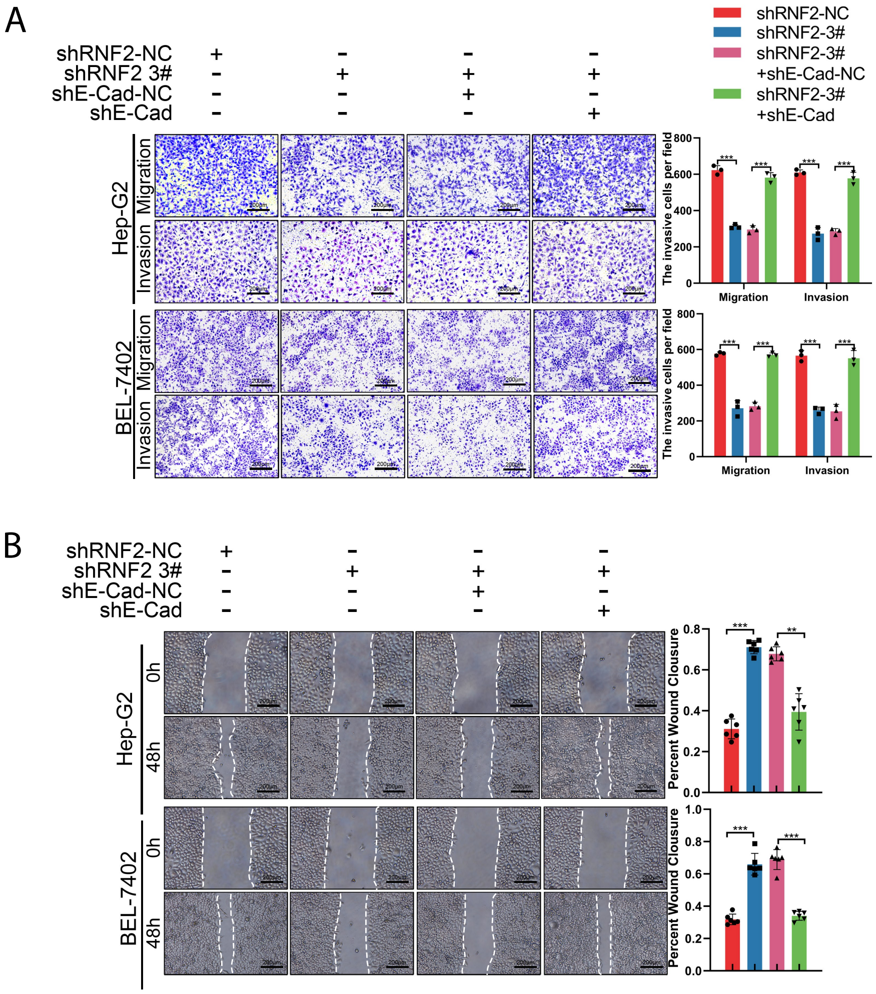

Supplementary Fig. S7 Representative images and quantitative analysis of Tanswell assay and Wound healing assay using transfected Hep-G2/BEL-7402.

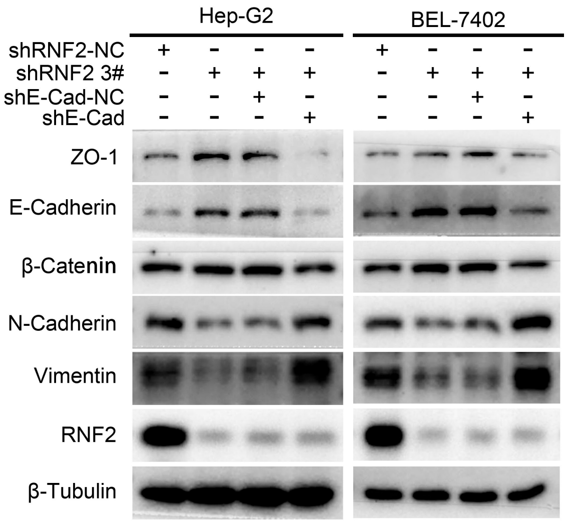

Supplementary Fig. S8 Western blot results of altered EMT-related protein expression after knockdown of RNF2 in Hep-G2/BEL-7402 cells and reversal of this alteration by co-knockdown of E-Cadherin.
