# Supplementary material for: RNF2 inhibits E-Cadherin transcription to promote hepatocellular carcinoma metastasis via inducing histone mono-ubiquitination
Source: Cell Death Dis. 2023 Apr 11;14(4):261. doi: 10.1038/s41419-023-05785-1 (PMC10085990; doi:10.1038/s41419-023-05785-1)
Supplement: Supplementary file 3 — Supplementary table 2 [file 41419_2023_5785_MOESM3_ESM.docx]

| **Antibody** | **Manufacturer** | **Cat. NO** | **Dilution** |
| --- | --- | --- | --- |
| RNF2 | Abcam | ab101273 | WB: 1:2000 |
| RNF2 | Proteintech | 16031-1-AP | WB: 1:1000 |
|  |  |  | IF: 1:200 |
|  |  |  | IHC: 1:200 |
| E-Cadherin | CST | #14472 | WB: 1:1000 |
|  |  |  | IF: 1:200 |
|  |  |  | IHC: 1:200 |
| ZO-1 | CST | #13663 | WB: 1:1000 |
| β-Catenin | CST | #8480 | WB: 1:1000 |
| N-Cadherin | Abcam | ab18203 | WB: 1:1000 |
|  |  |  | IHC: 1:200 |
| Vimentin | CST | #5741 | WB: 1:1000 |
| Snail | CST | #3879 | WB: 1:1000 |
| β-Tubulin | Servicebio | GB11017 | WB: 1:2000 |
| H2AK119ub | Abcam | ab193203 | WB: 1:1000  CHIP: 1:50 |
| H3K27me3 | CST | #9733 | WB: 1:1000 |
|  |  |  | CHIP: 1:50 |
| H3K4me3 | CST | #9751 | WB: 1:1000 |
|  |  |  | CHIP: 1:50 |
| NR2C2 | Perseus Proteomics | PP-H0107B-00 | WB: 1:1000 |
|  |  |  | CHIP: 1:50 |
| H2A | CST | #12349 | WB: 1:1000 |
| PCNA | Proteintech | 10205-2-AP | WB: 1:1000 |
| EZH2 | Proteintech | 21800-1-AP | WB: 1:1000 |
| MLL1 | Affinity | DF13551 | WB: 1:500 |
| Goat anti-Mouse HRP | Proteintech | SA00001-1 | WB: 1:10000 |
| Goat anti-Rabbit HRP | Proteintech | SA00001-2 | WB: 1:10000 |
| Goat Anti-Mouse H&L | Abcam | ab150116 (594) | IF: 1:200 |
| Goat Anti- Rabbit H&L | Abcam | ab150077 (488) | IF: 1:200 |

**Supplementary Table 2.** List of antibody and dilution ratio
