# Supplementary material for: RNF2 inhibits E-Cadherin transcription to promote hepatocellular carcinoma metastasis via inducing histone mono-ubiquitination
Source: Cell Death Dis. 2023 Apr 11;14(4):261. doi: 10.1038/s41419-023-05785-1 (PMC10085990; doi:10.1038/s41419-023-05785-1)
Supplement: Supplementary file 4 — Supplementary table 3 [file 41419_2023_5785_MOESM4_ESM.docx]

| **Gene** | **Primer sequences** |
| --- | --- |
| RNF2-Forward | AGGCCAGACCCAAACTTTGATGC |
| RNF2-Reverse | ATGCTGTGACTGAGTGCTTGCTG |
| E-cadherin-Forward | ATTTTTCCCTCGACACCCGAT |
| E-cadherin-Reverse | TCCCAGGCGTAGACCAAGA |
| NR2C2-Forward | TCCCCACGCATCCAGATAATC |
| NR2C2-Reverse | GATGTGAAAACACTCAATGGGC |
| TCFL5-Forward | CACAGCATTCCTGAAATACATCC |
| TCFL5-Reverse | GTCTGGTCAGCTTTAGCCT |
| ZNF740-Forward | GCAGGTGTGAGTTTGGTTCC |
| ZNF740-Reverse | CCTCAGCACATCAGGGCTAC |
| SP2-Forward | TCTGCCCGTCAACAACC |
| SP2-Reverse | CGATCAGCACCGTCTCC |
| HES7-Forward | CGGGATCGAGCTGAGAATAGG |
| HES7-Reverse | GCGAACTCCAATATCTCCGCTT |
| HNF4G-Forward | ATGGACATGGCAAATTACAGTGA |
| HNF4G-Reverse | TTGACACCGTTGTCTGTGGTA |
| GAPDH-Forward | CAGGAGGCATTGCTGATGAT |
| GAPDH-Reverse | GAAGGCTGGGGCTCATTT |

**Supplementary Table 3.** The List of primer sequences involved in qPCR
