# Supplementary material for: RNF2 inhibits E-Cadherin transcription to promote hepatocellular carcinoma metastasis via inducing histone mono-ubiquitination
Source: Cell Death Dis. 2023 Apr 11;14(4):261. doi: 10.1038/s41419-023-05785-1 (PMC10085990; doi:10.1038/s41419-023-05785-1)
Supplement: Supplementary file 5 — Supplementary table 4 [file 41419_2023_5785_MOESM5_ESM.docx]

**Supplementary Table 4.** The List of shRNA target sequence involved in RNAi

| **Gene** | **Target Sequence** |
| --- | --- |
| shRNF2-1# | GCTGTGAGGTTAGCTTTAGAA |
| shRNF2-2# | CGAAGTCTACACAGTGAATTA |
| shRNF2-3# | GCCAGGATCAACAAGCACAAT |
| shE-Cadherin | GAACGAGGCTAACGTCGTAAT |
| shNR2C2 | GGCTGATGAGCTCCAACATAA |
| shTCFL5 | GAATCCACTAAACAGACGTTA |
| shSP2 | GGACCCGATCAAATGCCAATA |
| shHES7 | CAAGATGCTCAAGCCGCTTGT |
| shHNF4G | GCATTCGTAAGAGTCACGTTT |
| shZNF740 | GCTGCTCAAGAAACAAAGGAA |
