# Supplementary material for: RNF2 inhibits E-Cadherin transcription to promote hepatocellular carcinoma metastasis via inducing histone mono-ubiquitination
Source: Cell Death Dis. 2023 Apr 11;14(4):261. doi: 10.1038/s41419-023-05785-1 (PMC10085990; doi:10.1038/s41419-023-05785-1)
Supplement: Supplementary file 6 — Original data files [file 41419_2023_5785_MOESM6_ESM.pdf]

Figure 1 F

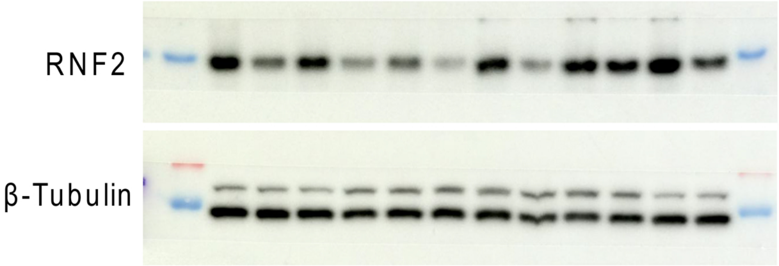

Figure 2 B

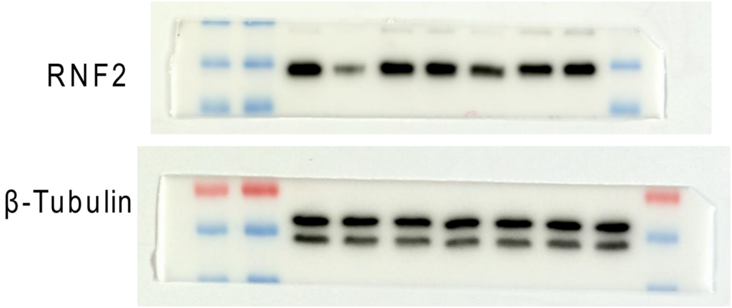

Figure 1 H

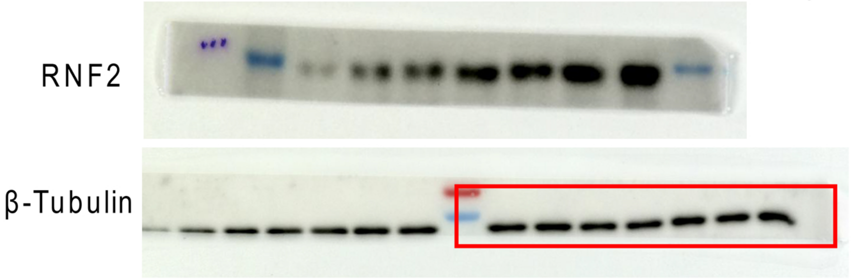

Figure 3 E

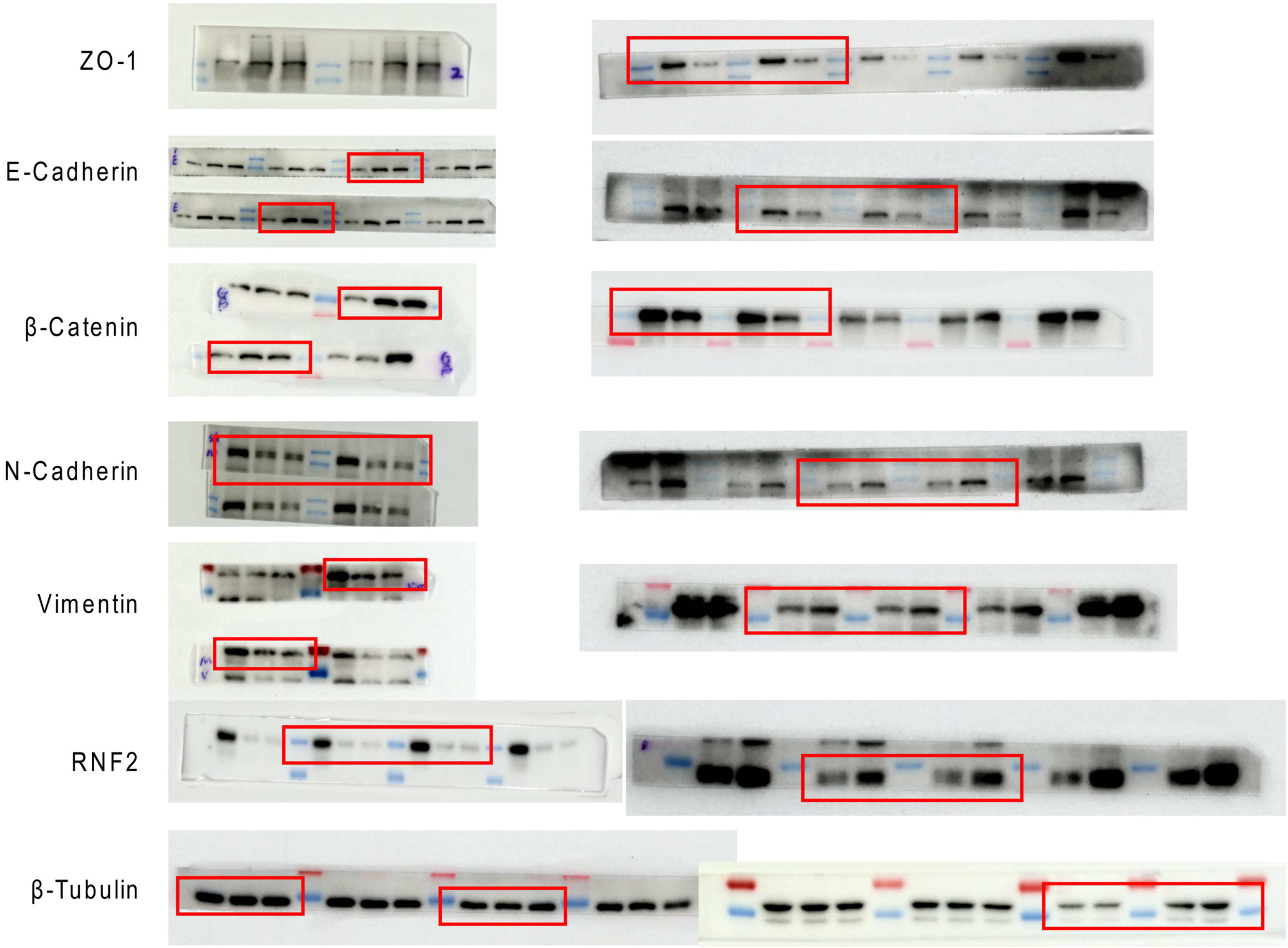

Supplementary Fig. S9 Original blot of Fig. 1F, H, Fig. 2B and Fig 3E.

Figure 4 E

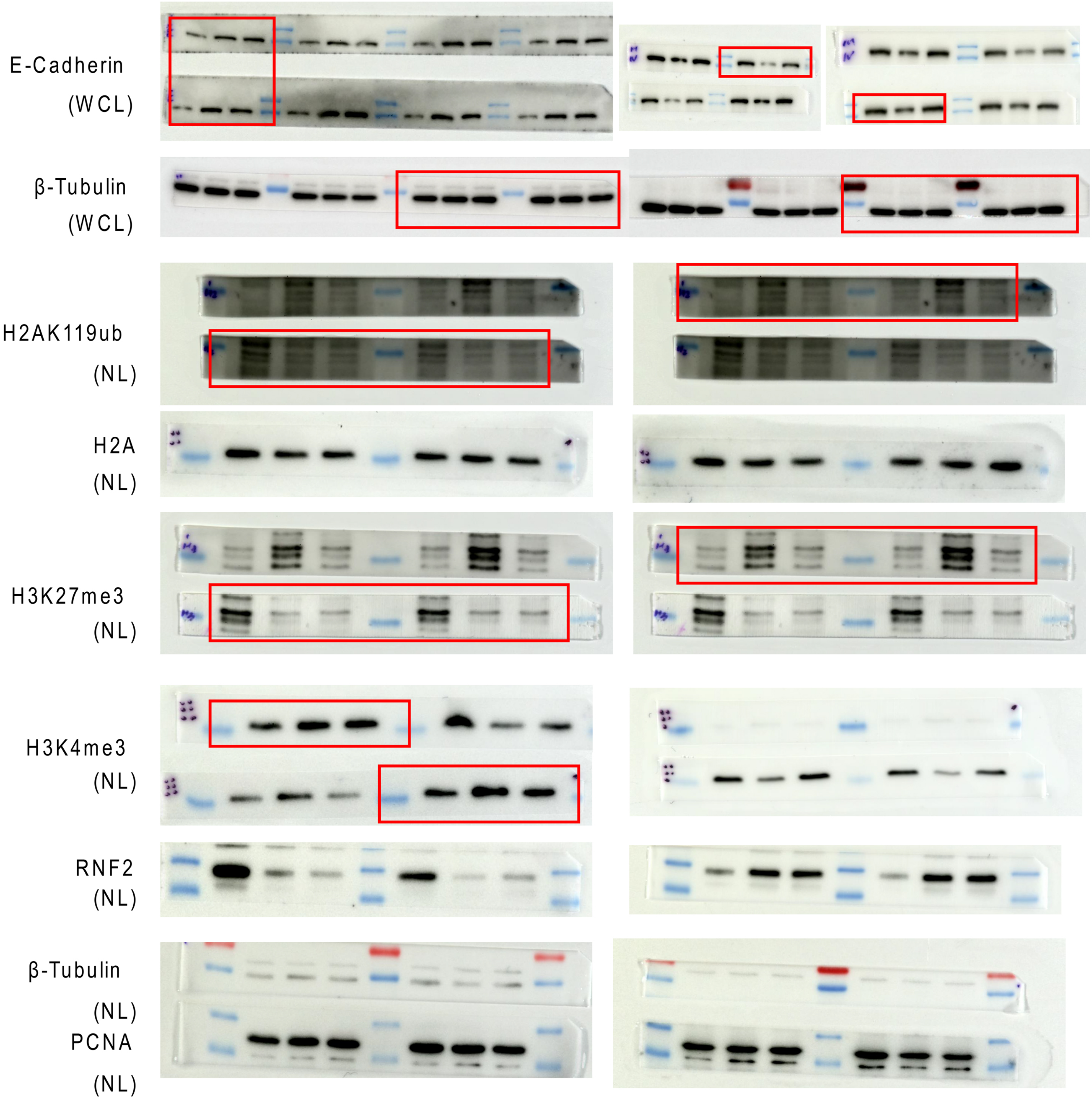

Supplementary Fig. S10 Original blot of Fig. 4E.

Figure 4 L

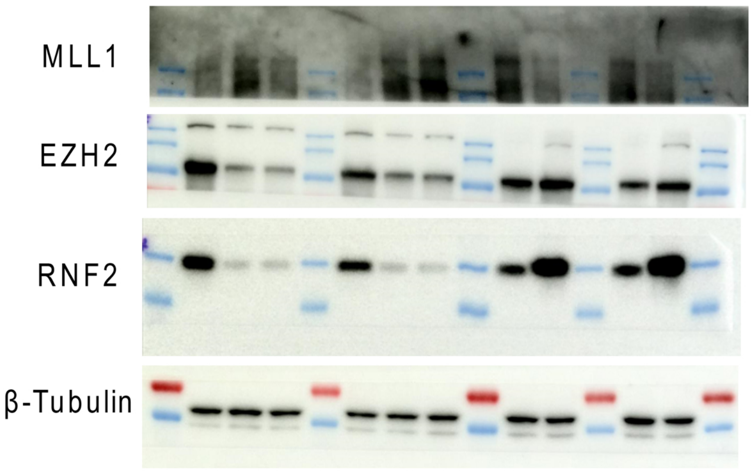

Figure 4 M

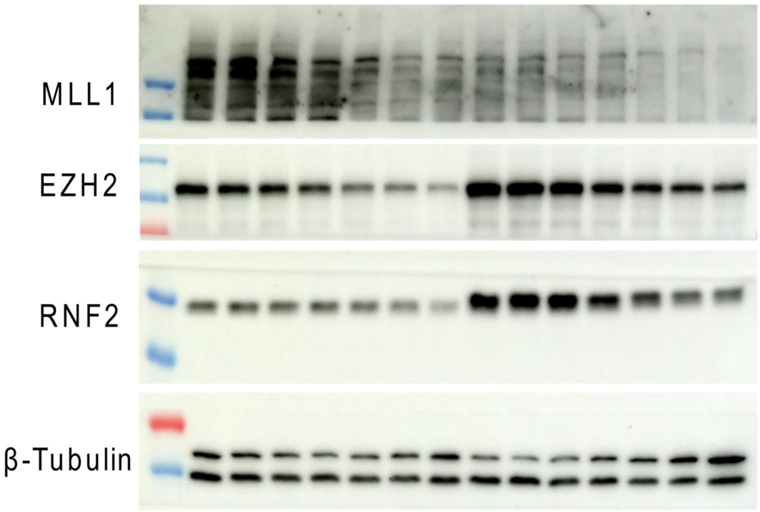

Figure 5 E

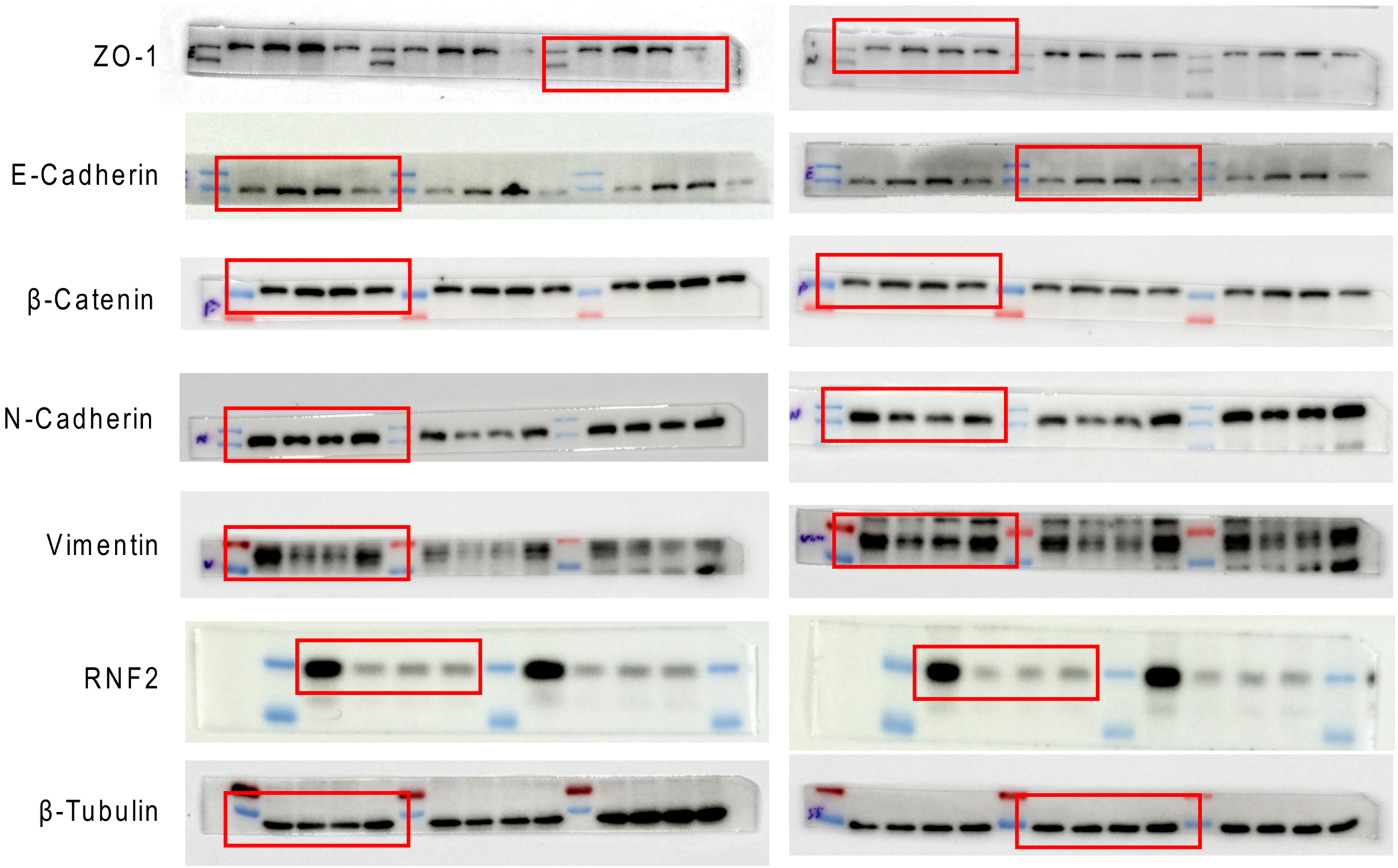

Supplementary Fig. S11 Original blot of Fig. 4L, M and Fig 5E.

Figure 5 F

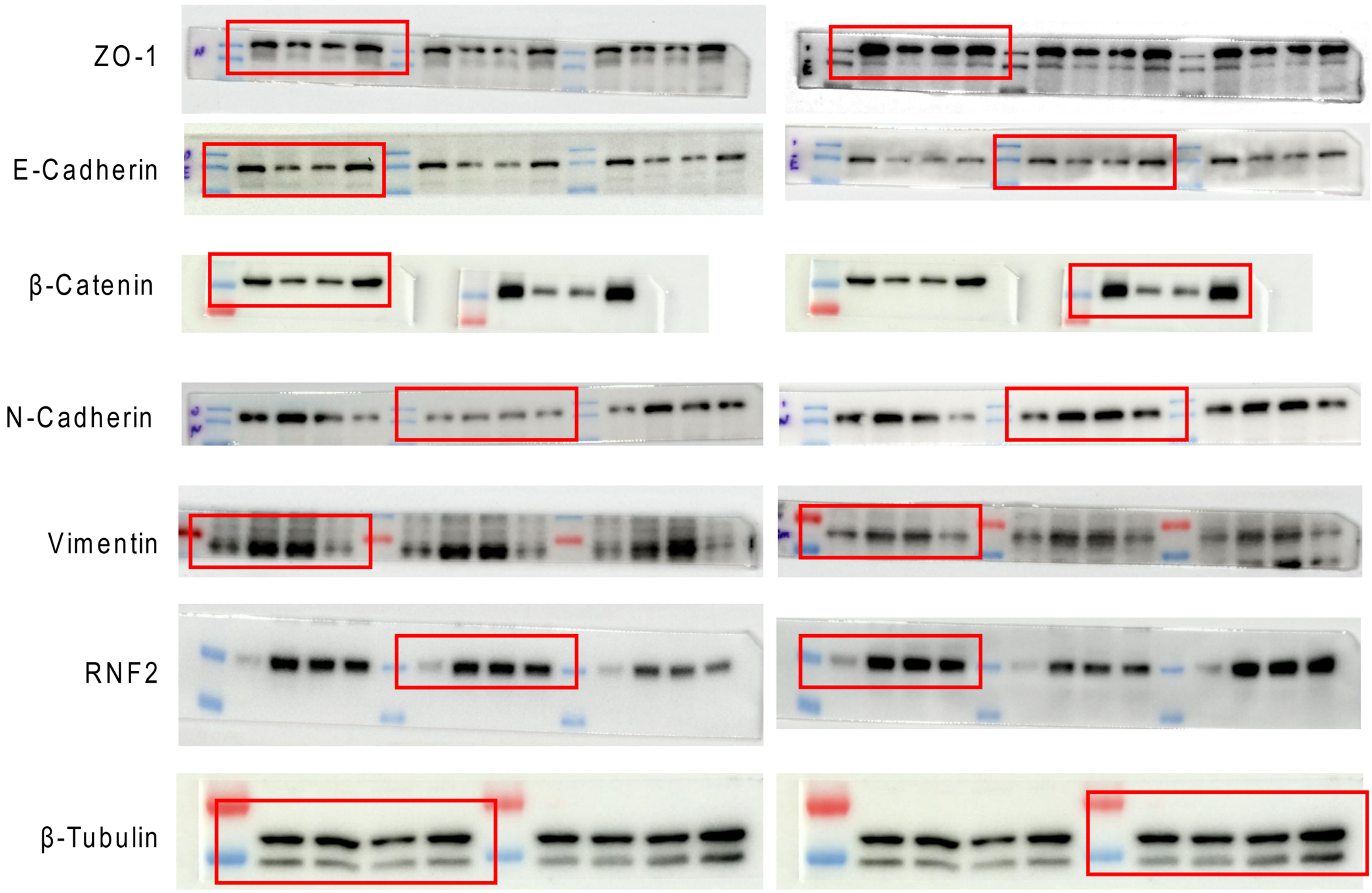

Supplementary Fig. S12 Original blot of Fig. 5F.

Supplementary Figure S1B

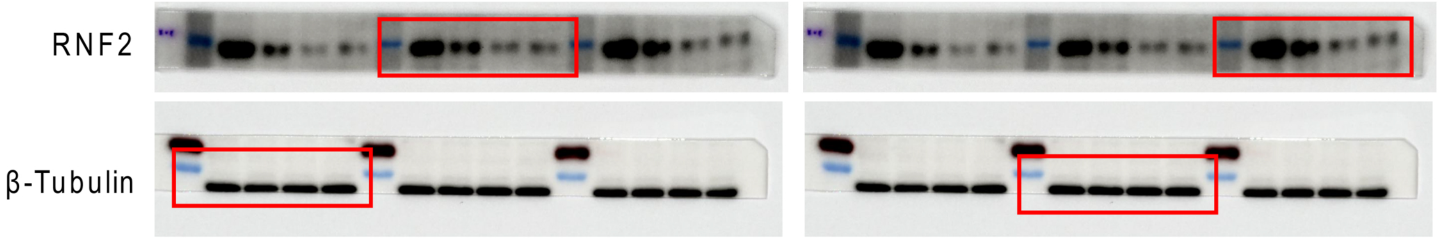

Supplementary Figure S1D

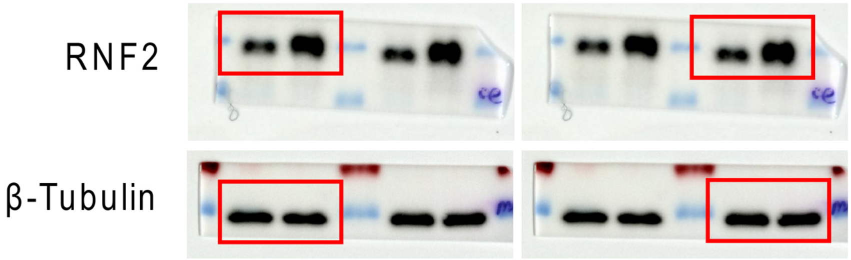

Supplementary Figure S3

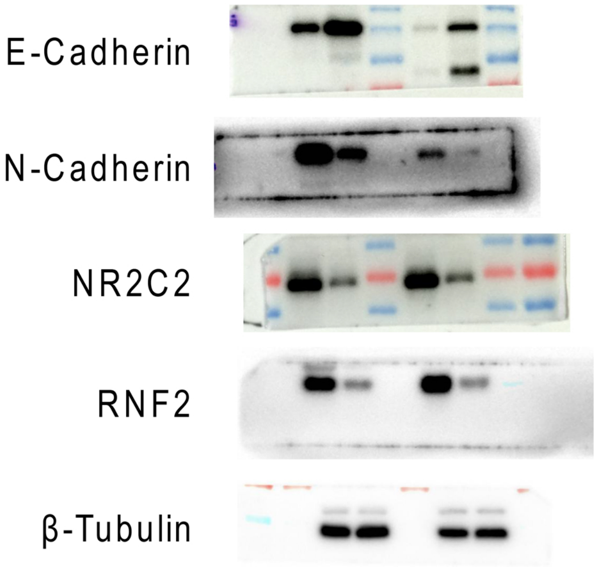

Supplementary Figure S5

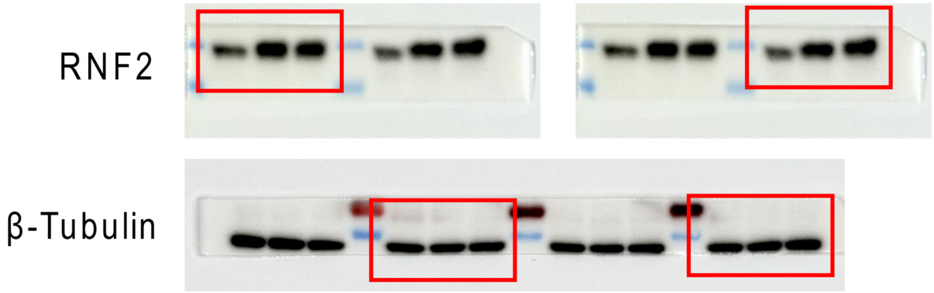

Supplementary Fig. S13 Original blot of Supplementary Fig. S1B, D, S3, S5.

Supplementary Figure S8

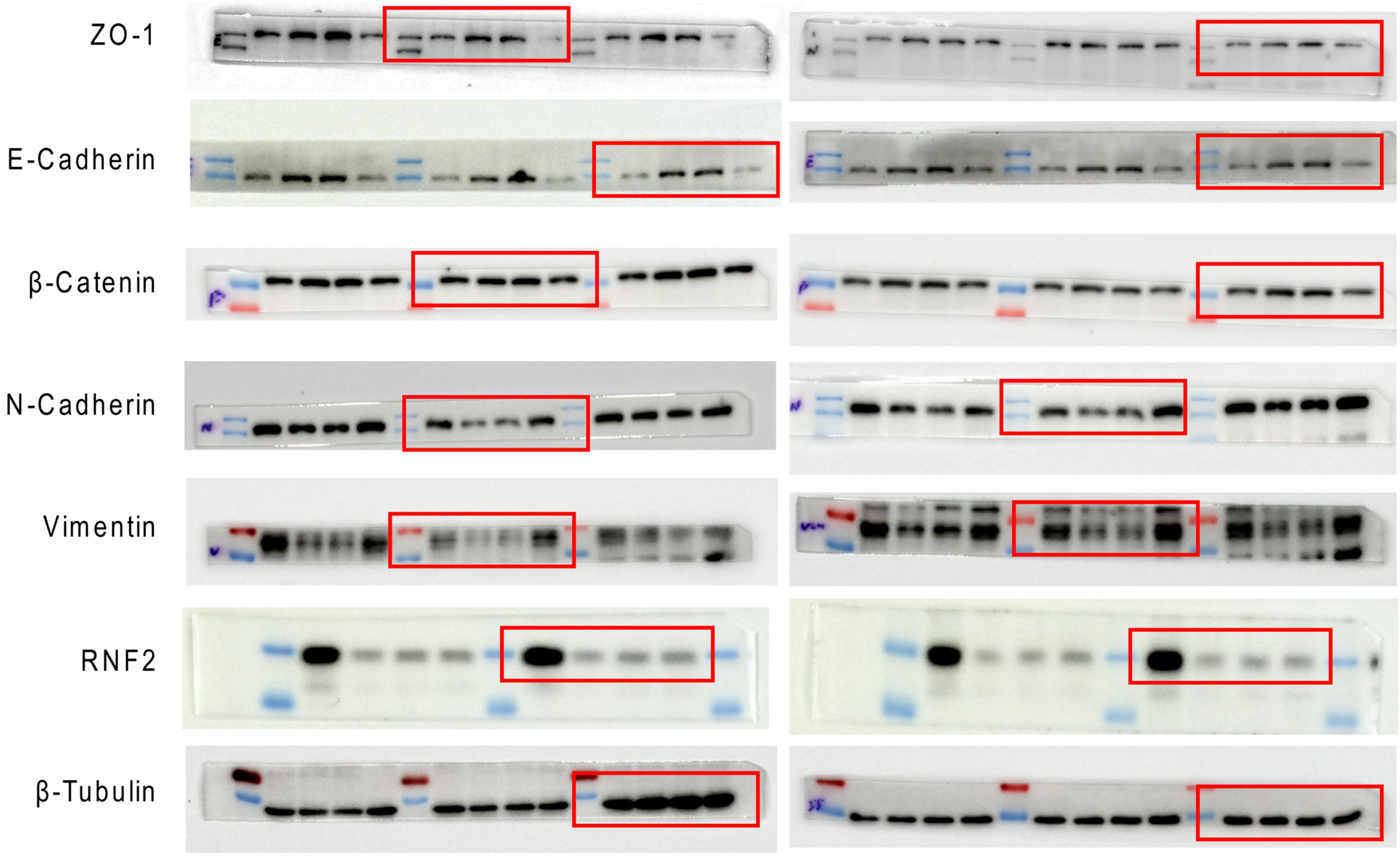

Supplementary Fig. S14 Original blot of Supplementary Fig. S8.
