# Supplementary material for: RNF2 inhibits E-Cadherin transcription to promote hepatocellular carcinoma metastasis via inducing histone mono-ubiquitination
Source: Cell Death Dis. 2023 Apr 11;14(4):261. doi: 10.1038/s41419-023-05785-1 (PMC10085990; doi:10.1038/s41419-023-05785-1)
Supplement: Supplementary file 7 — Author Contribution Statement [file 41419_2023_5785_MOESM7_ESM.docx]

**Author contribution Statement**

The study was designed by GZ, ZW and XL. LY preformed experiments and wrote the manuscript. ZZ analisised the data. JL assisted in providing clinical biological samples. JB, XO, YX and GL provided technical assistance. The manuscript was revised by all authors.
